# Non-Thermal Treatment Mediated by Curcumin for Enhancing Food Product Quality

**DOI:** 10.3390/foods13233980

**Published:** 2024-12-09

**Authors:** Ziyuan Wang, Haihong Yang, Zhaofeng Li, Jie Liu

**Affiliations:** 1 Key Laboratory of Geriatric Nutrition and Health, Beijing Technology and Business University, Ministry of Education, Beijing 100048, China; wangziyuan@btbu.edu.cn; 2 National Center of Technology Innovation for Grain Industry, Comprehensive Utilization of Edible By-Products, Beijing Technology and Business University, Beijing 100048, China; yanghaihong9811@163.com; 3 School of Food Science and Technology, Jiangnan University, Wuxi 214122, China; zfli@jiangnan.edu.cn

**Keywords:** non-thermal treatment, curcumin, food security, food quality

## Abstract

Increasing antibiotic resistance is one of the world’s greatest health problems, and biocide use in food disinfection, alongside other application fields, could increase antibiotic resistance. Effective and eco-friendly food decontamination treatment with minimal chemical intervention in food production is urgently needed. Synergistic antimicrobial interaction of photoactive compounds and blue-light-emitting diodes have recently been proven effective in agricultural and environmental applications. Curcumin-based non-thermal treatment has been reviewed in this work for the development of a safe and effective decontamination tool that could be adapted to the food industry. The antimicrobial mechanism of the synergistic interaction and the inhibitory efficacy against foodborne pathogens (bacteria in both vegetative form and spore, as well as in biofilms) are discussed. Further studies on curcumin and its derivative, as well as light illumination patterns, were compared for enhanced bactericidal efficacy. Moreover, studies relating to photodynamic inactivation treatment for food sanitation and food quality enhancement (cereal grains and other food products) were summarized, as well as the impact on food organoleptic and nutritional quality.

## 1. Introduction

Nowadays, the outbreak of foodborne diseases has become a crucial issue that threatens food safety and public health [1,2]. Due to incomplete sterilization or improper preservation, microorganisms in food could multiply quickly, resulting in food spoilage and deterioration, along with serious economic losses [3]. Therefore, exploring safe, environmentally friendly, and efficient means for controlling and eradicating harmful microorganisms has always been a concern for both researchers and consumers [4]. However, long-term use of chemical preservatives has induced antibiotic-resistant microorganisms in the environment, creating new challenges for the fresh-produce market, while the existing disinfection strategies are not efficient [5]. Non-thermal food decontamination technologies, such as ultrasound, pulsed electric field (PEF), ionizing radiation, and cold plasma (CP), have received increasing attention recently [6]. However, there are still concerns about the practical application of these technologies due to the low sterilization efficiency and the safety hazards they pose [4,7,8,9]. For instance, PEF has a limited spore inactivation efficiency and may cause structural changes [10]. And CP-treated food might induce the formation of toxic compounds [6]. Furthermore, microbial resistance to PEF and ultrasonication have been reported [11], reinforcing the urgent need for an alternative disinfection technique that is safe, efficient, and cost-effective.

Photodynamic inactivation (PDI) is a new and promising strategy for eradicating microorganisms. It involves using a photosensitizer, light, and oxygen to damage and kill cells, especially harmful ones like microorganisms [12]. PDI sterilizes food products through the synergistic interaction between photoactive compounds (photosensitizers, PS) and visible-light illumination, and it is a safe, effective, and environment- and human-friendly method [13]. Photosensitization, which does not adversely affect food texture and avoids the thermal effects associated with PEF, is currently becoming a focal point in the field of light-based food processing [2]. As a non-thermal disinfection technique, the bactericidal efficacy is attributed to the generated reactive oxygen species (ROS) by non-toxic photosensitizers (PS) upon light excitation in the presence of oxygen. The production of ROS could attack cellular components simultaneously, including nucleic acids, lipids, and proteins, causing cell death ultimately [13]. Existing non-thermal treatment methods still have some challenges, such as being time consuming, expensive, stimulating microbial resistance, and having adverse effects on food quality (e.g., color, texture, and bioactive compounds), thus limiting their commercial application in food processing [14]. Compared to other antimicrobial methods, photosensitized sterilization is economical, controllable, stable, and more efficient without affecting food texture [2,9,13]. One of the most important advantages of this multi-targeted strategy over other antibacterial approaches is no microbial resistance, making it gain increasing attention [15].

For the application in food processing, the ideal photosensitizer should have the characteristics of being natural, cost-effective, having good efficacy in low concentration, and being safe for human consumption [12]. Curcumin (CUR), as a yellow phenol compound extracted from the rhizome of turmeric, has been used as a food additive since ancient times, with low-cost, safe, non-toxic, and non-polluting properties [12,16]. Presently, numerous studies have illuminated the biological features and medicinal properties of CUR, such as antioxidant, anti-cancer, anti-inflammatory, anti-diabetic, hypolipidemic, and other pharmacological benefits [17,18,19]. CUR is a natural photosensitizer that absorbs light in the wavelength range of 400–475 nm [16]. Curcumin-mediated photosensitization targets a range of bacteria and fungi, such as *Staphylococcus aureus*, *Enterococcus faecalis*, *Streptococcus mutans*, *Salmonella typhimurium*, *Streptococcus pyogenes*, *Lactobacillus* spp., and *Escherichia coli* [20]. Its combination with LED light sources has been shown to be useful in the purification of fresh foods, such as aquatic products, vegetables and fruits, dairy products, and poultry [2]. Moreover, the conjugation of CUR with edible biopolymers has yielded good results in the development of anti-microbial food packaging [14]. Its application in food ingredient production, product packaging, and other fields can help to enhance food microbiological control. In turn, it extends the shelf life of food, maintains the sensory and nutritional properties of food, and reduces foodborne diseases [13].

Therefore, the aim of this review is to present the synergistic potential of curcumin and light-emitting diodes for food sanitizing and cleaning labeling solutions. Starting from the bactericidal mechanism and mode of action of photosensitization, curcumin-mediated photodynamic inactivation in food packaging, processed surfaces, and food model systems is presented. This innovative approach opens a new and prospective avenue for the development of efficient, affordable, non-genotoxic, non-hazardous, and eco-friendly non-thermal antimicrobial techniques that may substitute for chemical interventions in food processing and agriculture.

## 2. Mechanisms of Antimicrobial Action

**Figure 1:**
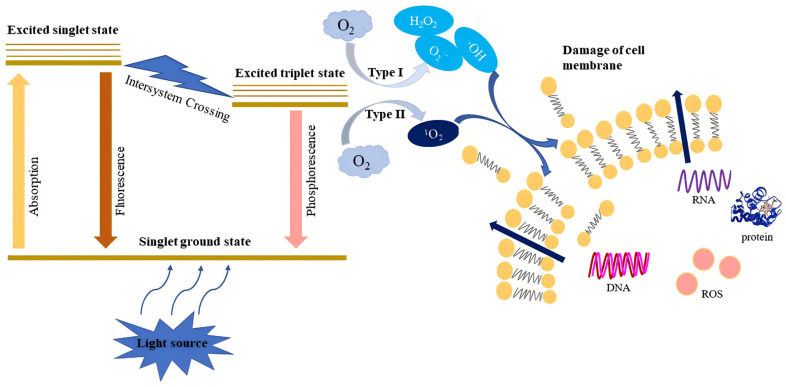
Schematic diagram of photosensitization processes.

In general, photosensitization is based on the activation of photosensitizers by light at an appropriate wavelength, along with the presence of oxygen molecules. The schematic diagram of the photosensitization processes is illustrated in Figure 1. The ground state photosensitizers are able to absorb the quantum of light and convert it to an excited singlet state, which is unstable and, further, can return to the ground state via releasing energy (emitting fluorescence) or transform to an excited triplet state via intersystem crossing [2]. Then, the excited triplet state PS could decay to the ground state by emitting phosphorescence or reacting with an acceptor substrate (e.g., organic molecules) to release the extra energy, resulting in the production of radicals.

These free radicals can target oxygen molecules and generate reactive oxygen species (ROS), such as hydrogen peroxide (H_2_O_2_), hydroxyl radicals (HO^·^), and superoxide anions (^·^O_2_^−^) (Type I mechanism). The ROS formed by the above mechanism can attack hundreds of cells per minute and react with the sensitive groups of biological molecules, causing multi-targeted cell damage [2,21,22]. Alternatively, the energy of the excited triplet state PS can be transferred to an oxygen molecule producing highly reactive singlet oxygen (^1^O_2_), which is cytotoxic and is predominantly involved in antibacterial photosensitization (Type II mechanism) [22,23]. The ^1^O_2_ can target different biomolecules, including aromatic side chains of amino acids, sulfur-containing amino acids, nucleic acids, and unsaturated lipids [24]. Both mechanisms induced oxidative damage to the cell membrane and cellular integrity, which could cause cytoplasm leakage, ending up in cell death. This mode of multi-targeted action has been proven to be efficient against a variety of microorganisms (e.g., parasitic protozoa, bacteriophages, fungi, Gram-positive and Gram-negative bacteria in a vegetative form, spores, and biofilms) with no emerging of mutagenic risks, namely no existence of antibiotic resistance [25].

**Figure 2:**
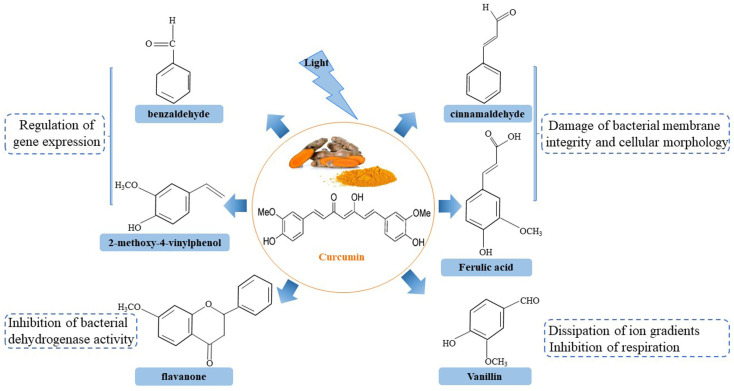
Photolysis products of curcumin and antibacterial mechanism.

In addition, the antimicrobial effect of photosensitization might be ascribed to the photolysis products of curcumin (Figure 2). Photolysis products, such as benzaldehyde and 2-methoxy-4-vinylphenol, could interfere with the normal expression of bacterial genes [20]. Cinnamaldehyde and ferulic acid derived from curcumin were found to disrupt cellular integrity and alter cellular morphology [26]. Vanillin was found to induce the dissipation of ion gradients and the inhibition of respiration. Flavanone was capable of inactivating bacteria by inhibiting their dehydrogenase activity, disturbing the normal physiological and biochemical reactions of the organism [27].

## 3. Light Sources and Irradiation Mode for Photosensitization

The light sources for the activation of photosensitizers include metal halide lamps, short-arc xenon, narrow-band UV lamps, and light-emitting diodes (LEDs). LED technology is based on semiconductors that emit selected ranges of wavelengths within the visible-light spectrum. Compared with UV illumination and conventional sources of light, LEDs, in terms of spectral properties and radiation pattern, show numerous advantages, including being environmentally friendly, energy-saving, safe, having a long lifespan, and being effective at causing a lethal photobiological response [2,24], without the concerns of harmful impacts on human health and deleterious effect on treated foods [28,29,30,31].

In general, the antimicrobial activity of LEDs is dependent on variable factors, such as the wavelength and irradiation mode. The present LED spectrum covers a wide range (250 nm–7 μm) and can be subdivided based on different wavelengths, among which blue LED (400–470 nm) was the most effective for microbial decontamination. Light irradiation can be performed in different manners, among them fractional or continuous. The continuous mode is traditionally used in PDT treatments, while the fractionation of the light dose is described in the literature as an advantageous protocol. This is because it allows for tissue reoxygenation during the period when it is not being illuminated, thus potentializing the PDT effect.

## 4. Bacteriostatic Action of Photosensitization

The effectiveness of synergistic interaction by CUR and LED on planktonic microorganisms has been summarized in Table 1. In this paper, the reduction of the number of bacterial colonies is evaluated by the logarithmic value of the total number of bacterial colonies per milliliter of sample (log CFU/mL). Both Gram-positive bacteria and Gram-negative bacteria were effectively inactivated by the combined treatment of CUR and blue-light (400–500 nm) illumination [32]. Aided by CUR (75 μM), a 3.57 log CFU/mL reduction of *Staphylococcus aureus* was achieved after 10 min of light exposure (139 J/cm^2^). Meanwhile, significant antimicrobial action against *Aeromonas hydrophila*, *Salmonella typhimurium*, *Pseudomonas aeruginosa*, and *Escherichia coli* was observed, with a corresponding reduction of 3.33, 1.26, 0.24, and 1.29 log CFU/mL, respectively. Similarly, Jiang et al. found that CUR (0–2.5 μM) and blue LED (3 J/cm^2^) reduced the growth of *S. aureus* by around 2.5 log CFU/mL [33]. Using CUR (5 μM) and blue light (3.6 J/cm^2^), Gao et al. found that the viability of *E. coli* DH5α decreased by 3.5 log CFU/mL in a dose-dependent manner [34]. Wu et al. [35] reported that the combined treatment of CUR (10 and 20 μM) and LED irradiation (470 nm, 3.6 J/cm^2^) resulted in a >6.5 log CFU/mL reduction of *Vibrio parahaemolyticus*. The bacterial death was partially induced by the ROS generated during PDI treatment, which disrupted the structure of the cell membrane, attacked intracellular amino acids, and broke down nucleotides. Najafi et al. [36] reported that *Aggregatibacter actinomycetemcomitans* was susceptible (inhibition of ~3 log) to CUR (2.5 mg/mL) and LED (420–480 nm, 120 J/cm^2^). Using CUR (3.7 mg/L) as PSs, a complete inactivation (6.1 log CFU/mL) of *Listeria innocua* was achieved after LED (400–500 nm) irradiation for 30 min [37]. It has been shown that treatment with 60 μM curcumin and blue light (430 nm, 33.01 J/cm^2^) could reduce 0.31 log CFU/mL and 1.39 log CFU/mL of *Pseudomonas lundensis* and *Brochothrix thermosphacta*, respectively [38]. Additionally, Saraiva et al. [39] found that curcumin combined with 450 nm light (0.81 J/cm^2^) in an aqueous solution inhibited 7 log CFU/mL of *Pseudomonas fluorescens*. Pang et al. [40] found that *Penicillium expansum* was completely inhibited by CUR (400 μM) and LED treatment in a liquid medium.

In addition to vegetative cells, the survival of spores is supposed to gain special attention in the food industry due to their high resistance to conventional sterilization approaches. Photodynamic inactivation has been reported to reduce >3 log CFU/mL of *Aspergillus flavus* spores by using 15–50 μM CUR and 84 J/cm^2^ illumination dose [41]. Al-Asmari, Mereddy, and Sultanbaw [42] also reported that CUR (600–1000 μM) and illumination (dose of 96 J/cm^2^) could reduce spores of *Aspergillus niger*, *Aspergillus. flavus*, *Penicillium griseofulvin*, *Penicillium chrysogenum*, and *Zygosaccharomyces bailii* by 78.1–99%. Furthermore, the inhibition ratio achieved 100% for *Fusarium oxysporum* and *Candida albicans* after CUR (800 μM) combined illumination treatment (dose of 360 J/cm^2^). The effect of photoinactivation partially relied upon the uptake and accumulation of CUR on the spore surface, which may be impacted by the chemical properties of the spore membranes. It has also been pointed out that PDI-induced ROS production and biochemical reactions could result in obvious abnormalities in spore morphology and ultrastructure, such as cell wall atrophy, cytoplasmic exocytosis, and nuclear membrane blurring [43]. These results indicated that CUR-mediated PDI could be applied as a valuable alternative sanitation technology against planktonic bacteria and even fungal spores.

**Table 1:** The parameters and effect of CUR-mediated PDI against foodborne pathogens.

Microorganism	Concentration of CUR	Wavelength	Energy Density	Irradiation Time/Dose	Reduction	References
*S. aureus ATCC 25923* *A. hydrophila ATCC 7966* *S. Typhimurium ATCC 14028* *P. aeruginosa ATCC 27853* *E. coli ATCC 25922*	75 μM	470 nm	678 mW/cm^2^	10–30 min 139, 278, 417 J/cm^2^	~3–4 log CFU/mL	[32]
*S. aureus*	0–2.5 μM	470 nm	60 mW/cm^2^	3 J/cm^2^	~2.5 log CFU/mL	[33]
*E. coli DH5α*	5, 10, 20 μM	470 nm	0.06 W/cm^2^	3.6 J/cm^2^	3.5 log CFU/mL	[34]
*V. parahaemolyticus* *ATCC 17802*	5, 10, 20 μM	470 nm	0.06 W/cm^2^	3.6 J/cm^2^	>6.5 log CFU/mL	[35]
*A. actinomycetemcomitans* *ATCC 33384*	2.5 mg/mL	420~480 nm	400 mW/cm^2^	120 J/cm^2^	~3 log CFU/mL	[36]
*L. innocua NCTC 11288*	3.7 mg/L	400~500 nm	150 mW/cm^2^	30 min	6.1 log CFU/mL	[37]
*P. Lundensis* *B. thermosphacta*	60 μM	430 nm	34 W	30 min 33.01 J/cm^2^	0.31 log CFU/mL 1.39 log CFU/mL	[38]
*P. fluorescens ATCC 13525*	62.5 mg/mL	450 nm	2.7 mW/cm^2^	5 min 0.81 J/cm^2^	7 log CFU/mL	[39]
*P. expansum*	400 μM	420 nm	50 W	20 min 330 J/cm^2^	100%	[40]
*A. flavus ATCC 28893*	5–100 μM	420 nm	NS	12–84 J/cm^2^	>3 log CFU/mL	[41]
*A. niger ATCC 6275* *A. flavus ATCC 9643* *P. griseofulvum ATCC 48927* *P. chrysogenum ATCC 10106* *F. oxysporum ATCC 62606* *C. albicans ATCC 10231* *Z. bailii ATCC 42476*	100–1000 μM	370–680 nm	NS	24–96 J/cm^2^	0.66–2.49 log CFU/mL	[42]
*P. italicum*	75 μmol/L	460 nm	200 mW/cm^2^	20 min	4.61 log CFU/mL	[44]

NS: not specified.

## 5. Photosensitization for Decontamination of Biofilms

The formation of biofilms adhering to food and various food-processing contact surfaces is an important cause of product contamination. Biofilm is a membranous multicellular complex, formed by self-encapsulation, attached to the surface of the host cavity or biological material. It can secrete polysaccharide substrates, lipoproteins, etc., which enhance resistance to sanitizers and sterilization procedures during food processing [22]. CUR-mediated PDI has been proven to be effective in controlling cross-contamination by biofilms in the food industry (Table 2).

Sanitá et al. [45] reported the inhibitory effect of CUR-mediated PDI against *Candida dubliniensis* and showed an 82.05% reduction for biofilm cultures. Similar results have been reported for biofilms of *Candida albicans*, *Candida Glabrata*, and *Streptococcus mutans* after CUR and blue-light treatment [46]. The authors found that 48 h biofilm was more resistant than 24 h biofilm, which was probably related to the increased amount of extracellular substance during the longer mature period [47]. The viability of *Candida albicans* biofilm decreased with the increase in the CUR concentration, and the highest PDI efficacy (87% reduction) was achieved when treated with a longer incubation time (20 min) before irradiation [48]. For *Enterococcus faecalis* biofilm, CUR (5.0 mg/mL) combined with LED (450 nm, 300–420 J/cm^2^) could result in a reduction of 68.5% [49]. Furthermore, with the irradiation of 14.4 J/cm^2^, a decrease of 80% for *P. aeruginosa* biofilm was reached when a CUR microemulsion (5 ppm) was combined with EDTA (125 ppm) [50]. Abdulrahman et al. [51] stated that extracellular polymeric substances (EPS), an essential component of *P. aeruginosa* biofilm, were reduced by CUR-mediated PDI. This, in turn, affected the stability of the biofilm, which led to cell death in the end. Meanwhile, PDI treatment was able to inhibit the expression of population-sensing genes (QS), disrupting the normal expression of the biofilm formation-related gene. It was also found that 75 μmol/L CUR combined with 250 mg/L Vc reduced *Penicillium italicum* biofilm by 3.23 log CFU/mL after blue-light (460 nm, 200 mW/cm^2^) irradiation for 20 min [44]. The study indicated that the reduction of biofilm was caused by the significant structural damage and morphological changes made by PDI.

**Table 2:** The parameters and effects of CUR-mediated PDI against biofilms.

Microorganism	Concentration of CUR	Wavelength	Energy Density	Irradiation Time/ Dose	Reduction	References
*C. dubliniensis*	20, 30, 40 μM	455 nm	22 mW/cm^2^	5.28 J/cm^2^	82.05%	[45]
*C. albicans ATCC 90028* *C. glabrata ATCC 2001* *S. mutans ATCC 25175*	80, 100, 120 μM	440–460 nm	22 mW/cm^2^	29 min 37.5 J/cm^2^	lower livability	[46]
*C. albicans ATCC 90028*	5, 10, 20 μM	440–460 nm	22 mW/cm^2^	1.32–26.4 J/cm^2^	lower livability	[48]
*E. faecalis* *ATCC 29212*	5.0 mg/mL	450 nm	NS	300–420 J/cm^2^	68.5%	[49]
*P. aeruginosa* *BCRC 12154*	5 ppm CUR 125 ppm EDTA	455 nm	16 mW/cm^2^	15 min 14.4 J/cm^2^	80%	[50]
*P. Italicum*	75 μmol/L	460 nm	200 mW/cm^2^	20 min	~2.3Log CFU/mL	[44]

NS: not specified.

## 6. Efficiency of Synergistic Interaction in Food Model

**Figure 3:**
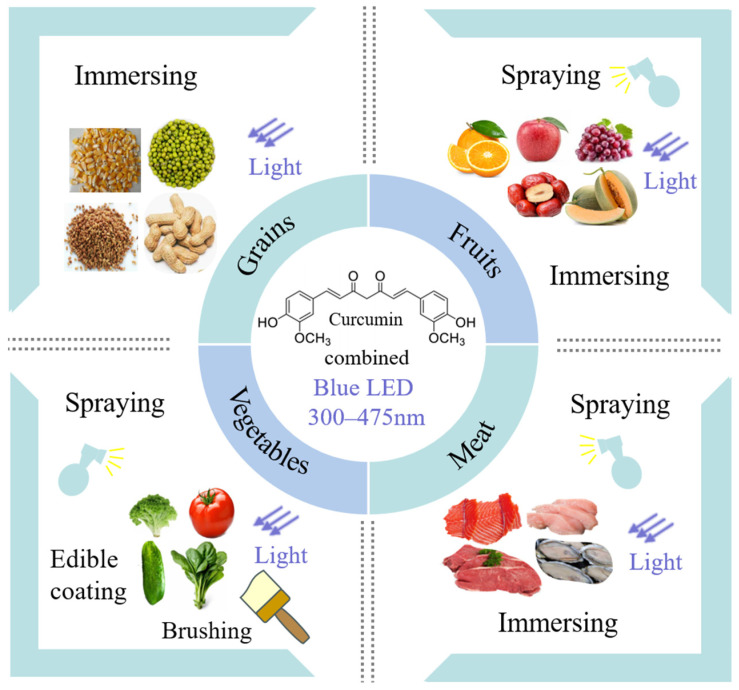
The practical application of CUR-mediated PDI in different food model systems.

Due to the increasing demands for a healthy diet, the tendency of consumption of aquatic products, grains, and fresh fruit and vegetables has increased. However, raw food materials have been known to be vehicles for the transmission of human diseases and have become the second leading cause of food-borne illnesses. Obviously, there is a need to develop novel processing technologies that are more effective and do not diminish the organoleptic properties and nutritional value of the treated foodstuffs. The practical application of CUR and LED in different food model systems has been listed in Table 3 and shown in Figure 3.

### 6.1. Fruits and Vegetables

Fresh fruits have high moisture content and sugar, which provides favorable conditions for the growth and reproduction of microorganisms. The current technology for fruit surface sanitizing involves chlorine water disinfection, high hydrostatic pressure treatment, electrolyzed oxidized water, ozonation, and X-ray irradiation [52]. Nevertheless, these methods are not fully applied to commercialization due to their limitations, while CUR-mediated PDI has been shown to be a promising alternative for the disinfection of fresh fruit without notable negative effects.

The fungal growth on the date surface was inhibited by spraying CUR solution on the date surface combined with blue-light illumination [52]. The shelf-life was prolonged from 7 days and 28 days to 14 days and 98 days for dates stored at 30 °C and at 4 °C, respectively, without quality loss, such as moisture content, total sugar, and total phenol content. Spoilage of fresh-cut Hami melons could be prevented by spraying curcumin solution (10–50 µM) and exposure to blue LED [53]. The combined treatment effectively reduced microbial growth by 1.8 log CFU/g, approximatively.

The antibacterial efficiency of CUR-mediated PDI against *E. coli* was verified on the surface of a fresh-cut “Fuji” apple, which was positively related to CUR concentration and irradiation time [54]. Additionally, the sensory and nutritional qualities of the fresh-cut apple were maintained during storage, including color, weight, ascorbic acid content, total phenolic content, and total antioxidant capacity. The enzyme activities of polyphenol oxidase and peroxidase were inhibited, which further prevented the unwanted browning of the fresh-cut apple. Aurum and Nguyen [55] showed that PDI could effectively inactivate ~2.4 log *E. coli* on the surface of a grape with exposure to blue light (36.3 J/cm^2^). Moreover, the photoactivation treatment largely preserved the original quality of the stored grape, including vitamin C, color, and firmness.

For the decontamination of orange juice, a combination of CUR and blue LED could inactivate *E. coli* and *S. aureus*, respectively [56]. Meanwhile, there was no significant change in the total phenolic, flavonoid, and hesperidin contents after the treatments. Additionally, Wang et al. [44] found that CUR-mediated PDI was able to reduce *Penicillium italicum* by 2.5 log CFU/mL in fresh-cut oranges. Photodynamic treatment with curcumin (0–75 μmol/L) had little effect on the color of the orange slices and also impeded the decrease in pH of the oranges during storage.

Fresh vegetables are highly susceptible to microbial contamination during processing and storage. The effect of CUR-mediated PDI on preventing microbial cross-contamination of fresh vegetables (spinach leaves and cherry tomato) has been verified [57]. Polyvinylpyrrolidone-CUR (PVP-C) has also been reported to cause a reduction of 2.6 log CFU/mL *S. aureus* in cucumber [58].

### 6.2. Cereal Grains

Grains are often contaminated with microorganisms and molds during harvesting, transportation, and storage, which seriously affects the quality and functional properties of grains [60]. Molds not only reduce the nutritional value of grains but also form mycotoxins and allergenic spores, which are the biggest spoilage organisms for grain quality [71]. Although new technologies such as cold plasma, irradiation, ozone, and biological methods have been developed for microbial safety, they still have limited applications [72,73]. Recently, non-thermal photosensitization has been proven to have good potential in grain storage and preservation.

It has been reported that the high-intensity light pulses generated by the LED can be used to reduce pathogenic bacteria in low-a_w_ foods [74]. For example, the maximum reduction of the *Salmonella* cocktail was 2.91 log after wheat flour was treated with a 395 nm pulsed LED for 60 min in a semi-closed system [75]. Similarly, the number of *Salmonella* in wheat flour was reduced by 1.07, 2.42, 3.67, and 2.64 CFU/g after treatments with 275, 365, 395, and 455 nm LEDs for 60 min at 25 °C and 75% relative humidity, respectively [76]. Not coincidentally, another study found that 5 h of LED blue-light treatment (430–470 nm, 36 W) on wheat flour with a high bacterial load could reduce the number of *Escherichia coli* colonies by 4.9 (lg (CFU/g)), while reducing the number of *Bacillus cereus* colonies by 4.7 (lg (CFU/g)), and the sterilizing effect was positively correlated with the duration of light exposure [77].

Additionally, the use of a photosensitizer-synergistic LED antimicrobial has a better sterilizing effect and a wider range of application scenarios. The photosensitizer riboflavin has potential antimicrobial, and antibiotic periplasmic activity against the rice leaf blight fungus (*Xanthomonas oryzae pv. oryzae*) [78]. In addition, CUR (25 μM) at a light dose of 4.32 J/cm^2^ produced significant photoinactivation of *S. saprophyticus* in vitro (approximately 5 log reduction), and there was a photo-antimicrobial effect on fresh millet dough (initial bacterial loads of 5 log CFU/g and 3 log CFU/g) of 1.04 log CFU/g and 0.81 log CFU/g [64]. Curcumin-based photosensitization has the advantages of being natural, green, economical, and effective in removing the aflatoxins that are commonly present in grain [79]. The effect of CUR-mediated PDI has also been evaluated for preventing the contamination of grains and seeds. It was found that CUR (1000 μM) and LED (430 nm, 104.2 J/cm^2^) photoinactivated *Aspergillus flavus* spores on maize kernels by 2.3 log CFU/mL and inhibited aflatoxin B1 (AFB1) production without affecting carotenoids [65]. A significant reduction (1.8–2.2 log CFU/g) of *A. flavus* spores on corn kernels was achieved after CUR (45 μM) and irradiation (420 nm) treatment [41]. Furthermore, the production of aflatoxin B_1_ in maize kernel was notably reduced from 305.9 μg/kg to 82.4 μg/kg after the CUR-mediated PDI treatment of *A. flavus* [66]. Nguenha et al. [67] reported that the photosensitization of ethanol and propylene glycol solubilized curcumin effectively and reduced the number of *Aspergillus flavus* on yellow and white corn kernel surfaces by 2.04 log CFU/mL and 3.33 log CFU/mL, respectively. Fungal growth in photosensitized maize kernels and flour was delayed by 14 and 7 days under 25 °C storage conditions. After 20 days of storage, AFB1 was not detected in maize kernels, and the amount of AFB1 in maize flour was reduced by 91%. Moreover, CUR (50 μΜ) and a 43.0 J/cm^2^ treatment inhibited the production of zearalenone (ZEN) by *F. graminearum*, contributing to a reduction from 1.15 mg/kg to 0.58 mg/kg. When the CUR concentration was ≥70 μM and the light energy was 43.0 J/cm^2^, *F. graminearum.* was completely inactivated [68].

Reductions of *E. coli* (~3 log CFU/mL) on fenugreek and mung beans were achieved when treated with the cationic CUR derivatives (SACUR-3) combined with blue LED (432 nm) and illumination (33.8 J/cm^2^) [69]. Additionally, CUR-mediated PDI was effective in mitigating the contamination of peanut seeds by *Aspergillus flavus* [70]. These results indicated that non-thermal treatment was a potential way to control the contamination of grains and seeds.

### 6.3. Animal-Based Food

Seafood, rich in nutritive value and biologically active ingredients, has aroused wide concern, as it is perishable at ordinary temperatures. Gao et al. reported that a 2.5 log reduction of *E. coli DH5α* in oysters was observed by using CUR combined with blue light [34]. And the PDI-treated oysters have been proven to be safe and nontoxic through cytotoxicity tests. Similarly, CUR solution sprayed on the surfaces of oysters following blue-light (470 nm) irradiation was found to extend the storage period from 8 days to 12 days stored at 5 ± 1 °C [80]. The PDI treatment improved oyster quality, lowered the oxidation degree, and slowed down the decomposition of nutrients. Chen et al. reported that CUR-mediated PDI was able to completely inhibit *Vibrio parahaemolyticus* on oysters when irradiated with blue light (455–460 nm, 9.36 J/cm^2^) [61]. PDI treatment inhibited the decrease in the pH value of oysters, reduced the production of TVB-N, and inhibited the lipid oxidation of oysters, prolonging the shelf-life of oysters.

CUR-mediated PDI has also been reported to be effective against the norovirus, which is one of the most crucial seafood-borne viruses and the leading cause of human acute gastroenteritis outbreaks [62]. A CUR solution (5, 10, 20 µM) combined with LED light (470 nm, 3.6 J/cm^2^) could cause a reduction of 1.15 log PFU/mL of norovirus in oysters by destroying the viral nucleic acids and capsid protein structure. Curcumin combined with 18.72 J/cm^2^ blue light inactivated ~3.0 log CFU/g of bacteria on salmon, achieving 99.9% bacterial inhibition. In addition, CUR-mediated PDI effectively inhibited salmon color and pH change and maintained muscle fiber integrity and SOD activity, preventing salmon protein and lipid degradation [61].

An investigation of PDI mediated by CUR was also conducted in meat products. In a beef model, PDI treatment decreased *Pseudomonas lundensis* and *Brochothrix thermosphacta* by 0.15 log CFU/g and 0.08 log CFU/g, respectively [38]. PDI treatment reduces the breakdown of proteins and lipids, reduces the production of undesirable odors, and maintains the bright-red color of beef.

Antimicrobial photodynamic protocols applying curcumin as a PS use a wide variety of parameters, and their efficiency in antimicrobial photodynamic therapy is related to the concentration they use and the species of microorganisms they target. The PDI of curcumin was effective in reducing the bacterial population, and its bactericidal effect was positively correlated with the duration of light exposure [2]. Considering the complexity of food ingredients, growth patterns of microorganisms, and practical use scenarios, it is difficult to compare the results of these descriptions using universal operational parameters [20].

## 7. Antimicrobial Effect of Curcumin Derivatives

In addition to the free form of curcumin, researchers have focused on developing new strategies to improve the physical–chemical properties of CUR by using micro-/nano-encapsulation and its derivatives [81]. These novel strategies have gained wide acceptance due to their advantages and application prospects over traditional materials. The anti-microbial activity of CUR derivatives is summarized in Table 4.

UNCPs-CUR synthesized by combining upconversion nanoparticles with CUR by using polyethyleneimine showed a complete inhibition (6 log CFU/mL) on methicillin-resistant *S. aureus* (MRAS) [82]. The combination of CUR-loaded cationic nanoparticles (CUR.NP) and LED illumination resulted in a decrease of >6.2 log of *Staphylococcus saprophyticus* and 2.9 log of *E. coli* [83]. A CUR/polyurethane nanocomposite was also found to inhibit *E. coli* and *S. aureus* (~6 log CFU/mL) completely after irradiation at 470 nm due to the phototoxicity and singlet oxygen produced [84]. Compared with the CUR solution, the synthesis of an antibacterial coating by using CUR in combination with blue-light illumination offered another new thought for preventing bacterial infection on food surfaces. Condat et al. [85] found that a CUR-derivative coating composed of epoxidized oil could achieve an inhibition rate of 99% and 95% against *S. aureus* and *E. coli*, respectively.

Microencapsulated CUR combined with blue LED achieved a good inhibitory effect against *P. aeruginosa* [50]. A complete photodynamic inhibition of *P. aeruginosa* was observed when a CUR microemulsion was combined with EDTA. Similarly, Hu et al. reported that the addition of EDTA significantly increased the antibacterial effect of CUR-mediated PDI against *Burkholderia cepaci* [86]. The bacterial inhibition rate was up to approximately 100% when treated with 50 μw CUR, including 0.4% EDTA following an irradiation dosage of 28 J/cm^2^. The reason might be due to the destruction of lipopolysaccharides in the outer membrane of Gram-negative bacteria caused by EDTA. In addition, NovaSol^®^–CUR was reported to sterilize *Staphylococcus aureus* in chicken meat, resulting in a reduction of 1.7 log [58].

Chen et al. prepared a 2,3-dialdehyde cellulose (DAC) antimicrobial membrane by adding a β-cyclodextrin/curcumin (β-CD/Cur) complex [87]. The film combined with PDI treatment was able to completely inhibit *L. monocytogenes* (>99.9%), *Vibrio parahaemolyticus* (>99.0%), and *Shewanella putrefaciens* (>99.9%). It is mainly the action of ROS (^1^O_2_, H_2_O_2_, etc.) produced by CUR after being excited by light and attacking intracellular proteins, nucleic acids, and lipids that kill bacteria.

## 8. Conclusions and Future Trends

The antimicrobial and antifungal activity of curcumin-mediated photodynamic inhabitation was reviewed, focusing on the inhibitory efficacy in vitro and in various food systems. Additionally, CUR in its free form and the derivatives with improved water solubility and chemical stability, including nano-/micro-formulations, polymeric micelles, and hydrogels, were evaluated. CUR-mediated PDI can also be used in combination with other physical sterilization methods to enhance the bactericidal effect, such as microwaves, ultrasound, pulsed light, and electron-beam irradiation. The large differences between different food matrices and compositions greatly increase the complexity of food sterilization and storage and preservation. Further research should focus on how PDI treatment affects the physicochemical properties and nutritional value of foods under actual food-processing conditions. In order to achieve the commercialized application of PDI for food sanitation and preservation, interdisciplinary research involving food science, microbiology, nutriology, and mathematical modeling is needed.

## Figures and Tables

**Table 3:** Antimicrobial activity of CUR-mediated PDI applied in food products.

	Foodstuff	Test Strains	CUR Concentration	Light Source	Irradiation Time /Light Dose	Reduction	References
Fruit	Fresh date fruit	NS	1000, 1400, 1800 μM	420 nm	10, 15 min 180, 270 J/cm^2^	shelf life extended by 7 days at 30 °C	[52]
	Hami melon	NS	10, 20, 40, 50 μM	460 nm	5, 30, 60, 90 min	~1.8 log CFU/g at storage	[53]
	Fresh-cut apples	*E. coli* *ATCC 25922*	0.5, 2, 10, 50 μM	298 mW/cm^2^	150–510 s	~0.95 log CFU/g	[54]
	Grape	*E. coli*	1600 μM	465–470 nm 4.5–30.2 mW/cm^2^	9.1, 18.1, 27.2, 36.3 J/cm^2^	0.95, 1.26, 2.18, 2.40 log CFU/g	[55]
	Orange juice	*E. coli* *ATCC 11775* *S. aureus* *ATCC 12600*	50, 100 μM	462 ± 3 nm	70 J/cm^2^	1.06 log CFU/mL 2.34 log CFU/mL	[56]
	Orange slices	*P. italicum*	75 μmol/L	460 nm 100 mW/cm^2^	20 min	~2.5 log CFU/g	[38]
Vegetable	Spinach leaves Cherry tomato	*E. coli O157:H7* *ATCC 700728* *L. innocua* *ATCC 33090*	5 mg/mL	320–400 nm 32 W/m^2^	5 min	4 log CFU/mL in wash water >100 CFU/cm^2^ on surface of product	[57]
	Cucumber	*S. aureus* *ATCC 25923*	50, 100 μM	435 nm 9.4 mW/cm^2^	33.8 J/cm^2^	2.6 log CFUs	[58]
	Spinach Lettuce Tomato	*E. coli O157:H7* *ATCC 700728* *L. innocua* *ATCC 33090*	10 mg/mL	320–400 nm 6.8 W/m^2^	5 min 20.4 kJ/m^2^	3 log CFU/mL 1.7–3.3 log CFU/mL	[59]
Seafood	Oysters	NS	10 μM	470 nm 0.06 W/cm^2^	90 s 5.4 J/cm^2^	storage time extended by 7 days at 4 °C	[60]
		*E. coli DH5α*	5, 10, 20 μM	470 nm 0.06 W/cm^2^	3.6 J/cm^2^	2.5 log CFU/g	[34]
		*V. parahaemolyticus*	100 μM	10 W, 455–460 nm	30 min 9.36 J/cm^2^	no visible colony was detectable	[61]
		*Norovirus*	5, 10, 20 μM	470 nm	3.6 J/cm^2^	1.15 log PFU/mL	[62]
Meat	Chicken meat	*S. aureus* *ATCC 25923*	50, 100 μM	435 nm 9.4 mW/cm^2^	33.8 J/cm^2^	1.7 log CFUs	[58]
	Sausages	*L. innocua* *ATCC 33090*	150 μg/mL	320–400 nm 32 ± 0.2 W/m^2^	30 min	>3 log CFU/mL	[63]
	Beef	*P. lundensis* *B. thermosphacta*	60 μM	34 W, 430 nm	33.01 J/cm^2^	0.15 log CFU/g 0.08 log CFU/g	[38]
Grains and Seeds	Millet dough	*S. saprophyticus*	25 μM	420–470 nm	4.32 J/cm^2^	~1 log CFU/g	[64]
	Corn kernels	*A. flavus spores* *aflatoxin B_1_*	1000 μM	430 nm	104.2 J/cm^2^	2.3 log CFU/mL inhibited aflatoxin B1 production	[65]
		*A. flavus spores*	0, 25, 45 μM	420 nm	60 J/cm^2^	~2 log CFU/g	[41]
		*aflatoxin B_1_*	50 μM	420 nm	60 J/cm^2^	production of aflatoxin B_1_ decreased significantly	[66]
		*A. flavus spores* *aflatoxin B_1_*	1000, 1250, 1500 μM	420 nm	60 J/cm^2^	storage time extended by 14 days at 25 °C inhibited aflatoxin B1 production	[67]
		*zearalenone*	50 μΜ	455~460 nm	43.0 J/cm^2^	ZEN decreased from 1.15 mg/kg to 0.58 mg/kg	[68]
	Corn flour	*A. flavus spores* *aflatoxin B_1_*	1000, 1500 μM	420 nm	60 J/cm^2^	storage time extended by 7 days at 25 °C inhibited aflatoxin B1 production	[67]
	Fenugreek Mung beans	*E. coli* *ATCC 25922*	10, 50, 100 μM	432 nm 9.4 mW/cm^2^	33.8 J/cm^2^	>3 log CFUs	[69]
	Peanut seeds	*A. flavus*	20 mg/mL	450 nm	NS	0.3 log CFU/g	[70]

NS: not specified.

**Table 4:** Investigations on photodynamic inactivation of CUR derivatives.

CUR Derivatives	Bacteria	Concentration	Light Source	Irradiation Time/Dose	Reduction	References
UNCPs-CUR	*S. aureus*	0, 10, 20, 40, 80, 320 µg/mL	980 nm 0.5 W/cm^2^	30 min	0–100%	[82]
CUR.NP	*S. saprophyticus* *DSM No. 18669* *E. coli* *DSM No. 6897*	NS	457 nm	105.6 J/cm^2^	>6.2 log CFU/mL ~2.9 log CFU/mL	[83]
CUR/polyurethane nanocomposites	*E. coli* *CCM 4517* *S. aureus* *CCM 4516*	NS	470 nm	1 h 6 h	100%	[84]
CUR derivative coatings	*S. aureus* *E. coli*	NS	420 nm	2, 6, 24, 48 h	99% 95%	[85]
Microemulsion combined with EDTA	*P. aeruginosa*	5 ppm CUR 15.4 ppm EDTA	455 nm 16 mW/cm^2^	15 min 14.4 J/cm^2^	90%	[59]
CUR combined EDTA	*B. cep aci*	50 μM CUR 0.4% EDTA	NS	28 J/cm^2^	100%	[86]
CUR-Zn	*P. aeruginosa (ATCC27853)* *E. coli O157:H7 (BNCC192101)* *P. Mirabilis (BNCC337267)* *E. coli (ATCC 25922)*	1 mg/mL	425 nm 6.25 mW/cm^2^	50 J/cm^2^	1.3 log CFU/mL 1.3 log CFU/mL 1.0 log CFU/mL 1.7 log CFU/mL	[70]
CUR-β-CD	*L. monocytogenes* *V. parahaemolyticus* *S. putrefaciens*	0.16 μM	10 W 455−460 nm	60 min 13.68 J/cm^2^	3.79 log CFU/g 2.81 log CFU/g 3.27 log CFU/g	[87]

NS: Not specified.

## Data Availability

No new data were created or analyzed in this study.
